# Return to Activity After Patellofemoral Osteochondral Fracture: A Comparison of Metallic Screw and Bioabsorbable Fixation

**DOI:** 10.1177/23259671241292641

**Published:** 2024-11-20

**Authors:** Kevin J. Orellana, Soroush Baghdadi, Daniel Yang, Julianna Lee, J. Todd Lawrence, Kathleen Maguire, Brendan A. Williams, Theodore Ganley

**Affiliations:** * Division of Orthopaedics, The Children’s Hospital of Philadelphia, Philadelphia, Pennsylvania, USA; † Department of Orthopaedic Surgery, Pediatric Perelman School of Medicine at the University of Pennsylvania, Philadelphia, Pennsylvania, USA; Investigation performed at The Children’s Hospital of Philadelphia, Philadelphia, Pennsylvania, USA

**Keywords:** pediatric sports medicine, osteochondral fractures, patellofemoral instability, general sports trauma

## Abstract

**Background:**

Patellofemoral osteochondral fractures (OCFs) have the potential to hinder patients’ function and quality of life. Several fragment fixation techniques have been described, with both metallic screw and bioabsorbable fixation showing favorable functional outcomes. Despite the promising results associated with both fixation methods, no study has directly compared their functional outcomes.

**Purpose:**

To compare the functional and radiographic outcomes between bioabsorbable and metallic screw patellofemoral OCF fixation in an adolescent cohort.

**Study Design:**

Cohort study; Level of evidence, 2.

**Methods:**

A retrospective review was conducted identifying surgically treated pediatric patients (<18 years of age) with OCFs of the patellofemoral joint. Inclusion criteria were treatment with metallic screw or bioabsorbable fixation (bioabsorbable compression screw, suture bridge, or chondral darts), with preoperative radiographs and operative notes available for review. Patient information, injury characteristics, treatments, and outcomes were collected with a specific focus on return-to-activity time and postoperative complications. Univariate analyses were conducted to compare radiographic and functional outcomes between groups.

**Results:**

According to the study criteria, 37 knees in 37 patients (84% male), with a mean age of 14.2 ± 1.8 years, were identified. A total of 24 patients were injured during sports participation, with basketball and football being the most common sports. OCF fixation cohorts consisted of 12 patients treated with metallic screw fixation and 25 with bioabsorbable fixation. No statistically significant differences were appreciated when comparing median time to full activity between the fixation groups (*P* = .427). However, time to full activity was unequally distributed, with 66.7% of the metallic screw fixation group returning to activity later than the total cohort’s median, compared with 42.9% of the bioabsorbable fixation group (*P* = .04). Two-thirds (8/12) of patients treated with metallic screws required return to the operating room for hardware removal compared with no patient treated with bioabsorbable fixation (*P* < .001). Two complications occurred with no significant differences appreciated between groups (*P* = .202). However, both postoperative complications were recorded in the metallic screw fixation group: 1 patient with osteochondral malunion and another with arthrofibrosis.

**Conclusion:**

This study demonstrated that pediatric patellofemoral OCFs had good outcomes with high healing and low complication rates regardless of fixation type. Because of the high rate of secondary hardware removal procedures, metallic screw constructs delayed the return to sports and activity time. Patients treated with bioabsorbable fixation did not require a secondary operation for hardware removal and thus were more likely to recover sooner. Future studies are necessary to assess the potential outcome differences between different types of bioabsorbable fixation methods. Based on these findings, surgeons can consider bioabsorbable fixation as an option for the management of OCF lesions.

Patellofemoral instability (PFI) is a common condition affecting pediatric and adolescent patients. The incidence of acute patellar dislocations is up to 77.4 per 100,00 patients per year, and their incidence has mirrored the steady increase in sports participation among children.^11,22^ Several risk factors for PFI have been identified, with female sex, trochlear dysplasia, and increased lateralization of the knee’s extensor mechanism being the most commonly cited in the literature.^19^ While the treatment of this condition has been rooted in addressing the anatomic risk factors that lead to recurrent instability, secondary injury to the underlying osteochondral joint surface adds complexity to the surgical decision-making for this condition.

Occurring in approximately 10% to 30% of first-time acute patellar dislocations, osteochondral fractures (OCFs) of the patella and lateral femoral condyle are a common complication associated with PFI.^7,16,18,21,23^ Given that osteochondral injuries such as OCF have been implicated in the development of osteoarthritis, it is of utmost importance to properly treat these injuries and preserve the function and long-term joint health in these young patients.^1^ Treatment of these injuries has largely relied on a variety of factors, including patient age, location, size, and displacement of the osteochondral fragment. Small or badly damaged fragments can be removed, but larger fragments can often be repaired.^4,5^ The vast majority of the displaced fragments are chondral fragments with only a small amount of bone; therefore, the primary goal of surgery is to stabilize the fragment to allow for healing of the chondral surface back to the subchondral bone. Despite recognition of the importance of fixation, no standardized method of fixation has been developed, with centers using a variety of bioabsorbable and nonbioabsorbable fixation methods, such as compression screws and absorbable pins.^2,24^

Recently, bioabsorbable fixation, including suture bridge fixation, has increased in popularity, with studies showing similar clinical outcomes to metallic nonabsorbable fixation, all while reducing the need for hardware removal and decreasing health care costs.^5,6,20^ Despite the potential for more widespread use of this fixation method, few studies have directly compared treatment outcomes between metallic and bioabsorbable fixation methods. Thus, the purpose of this study was to compare the functional and radiographic outcomes between metallic and bioabsorbable fixation for patellofemoral OCFs in an adolescent cohort. We hypothesized that bioabsorbable fixation, such as suture bridge and biocompression screws, would allow for similar radiographic healing to metallic screw fixation while decreasing the need for secondary surgical procedures.

## Methods

After approval from our center’s institutional review board (No. 15-012614), a retrospective review was conducted for all patients who sustained a patellar dislocation and were treated for OCFs of the patella or distal femur between 2012 and 2022. As both screw and suture bridge fixation typically require an open approach, Current Procedural Terminology (CPT) codes for open treatment of patellar and femoral fractures (CPT codes 27514 and 27524) were utilized as an additional search filter. Inclusion criteria for the study were patients <18 years of age who had imaging-confirmed OCFs after patellar dislocations and were treated by fragment reduction and fixation. Patients who did not meet the above criteria, those who were treated nonoperatively, or those who had other osteochondral lesions such as osteochondral dissecans were excluded from the review.

Clinical and operative records were reviewed by hand, and data was abstracted to collect patient and clinical information such as mechanism of injury, associated injuries, and pre- and postoperative range of motion (knee flexion and extension). Pre- and postoperative imaging were reviewed to evaluate skeletal maturity as determined by the status of the distal femoral physis, as well as fragment characteristics, and postoperative healing as defined by osteochondral integration. Operative records were specifically reviewed to obtain lesion location, size, and main fixation method utilized. The area was calculated as pi × length radius × width radius. Secondary surgical procedures, including those to address underlying PFI or other injuries, arthroscopic second look, and hardware removal, were also evaluated. Postoperative arthrofibrosis was defined as a 10° of extension deficit and/or a 25° of flexion deficit 3 months after surgery. Additionally, we evaluated the time for return to sports or full activity in those who did not participate in competitive sports, the rate of hardware removal, and the presence of complications.

Descriptive statistics were created for all variables, with continuous variables presented as mean with standard deviation for normally distributed data and median with range for nonnormally distributed data and categorical variables presented as counts and percentages. Univariate analysis was conducted comparing metallic screw and bioabsorbable fixation constructs using independent-sample Student *t* tests and analysis of variance for normally distributed continuous variables, the Mann-Whitney *U* test for nonnormally distributed continuous variables, and chi-square and Fisher exact tests for categorical variables. All statistical analysis was done utilizing SPSS statistical software (Version 28.0; IBM Corp) with an alpha value of 0.05.

## Results

The final cohort consisted of 37 OCFs in 37 patients (31 male, 6 female) with a mean age of 14.2 ± 1.8 years. The mean BMI was 24.8 ± 6.5 kg/m^2^. A total of 24 (64.9%) injuries occurred during sports participation, with football and basketball being the most common. Seventeen (45.9%) patients were evaluated with at least 1 associated knee injury. In total, there were 3 injuries to the lateral menisci, 1 medial meniscal tear, and 1 anterior cruciate ligament tear. The mean preoperative knee flexion was measured at 90.3°± 37.2° (range, 15° to 140°), while extension was measured at –2.9°± 11.4° (range, –45° to 20°). Cohort and injury characteristics are summarized in Table 1.

**Table 1:** Patient and Injury Characteristics^*a*^

Characteristic	Full Cohort (N = 37)	Metallic Screw (n = 12)	Bioabsorbable Fixation (n = 25)	*P*
Sex				
Male Female	31 (84) 6 (16)	11 (92) 1 (8)	20 (80) 5 (20)	.641
Age, y	14.2 ± 1.8	14.5 ± 1.9	14.0 ± 1.7	.423
BMI, kg/m^2^	24.8 ± 6.5	25.8 ± 2.5	24.2 ± 5.3	.50
Sport during injury				
Basketball Football Soccer Baseball/softball Other Nonsport injury	5 (14) 5 (14) 2 (5) 3 (8) 9 (24) 13 (35)	3 (25) 2 (17) 0 (0) 0 (0) 1 (8) 6 (50)	2 (8) 3 (12) 2 (8) 3 (12) 8 (32) 7 (28)	.214
Physeal status				
Open Closing Closed	19 (51) 14 (38) 4 (11)	4 (33) 7 (58) 1 (8)	15 (60) 7 (28) 3 (12)	.239
Patellar dislocation during injury	22 (59)	9 (75)	13 (52)	.462

a Data are presented a n (%) or mean ± SD. BMI, body mass index.

**Figure 1.**
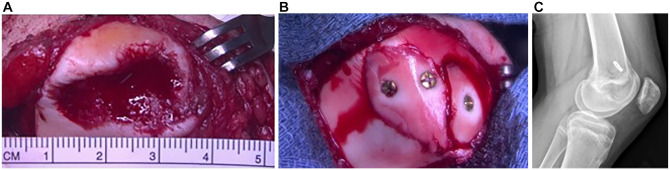
Image of the medial facet of a patellar osteochondral fracture. (A) Site of osteochondral fracture measuring 25 mm in diameter. (B) Osteochondral fragment reduced into lesion site and fixated with 3 headed mini-fragment metallic screws. (C) Lateral radiograph showing screw removal and medial patellofemoral ligament reconstruction that were done 6 months after initial injury, with intact and stable articular cartilage.

**Figure 2.**
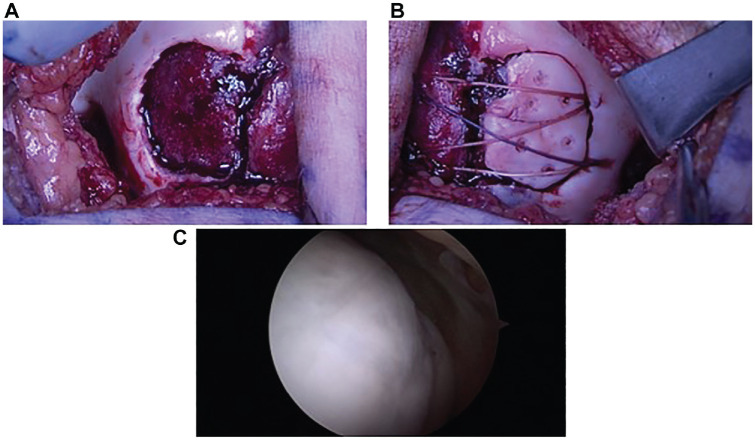
Image of a left lateral condyle osteochondral fracture. (A) Site of osteochondral fracture measuring 25 mm in diameter. (B) Osteochondral fragment reduced into lesion site and fixated with suture bridge and poly-l-lactic acid tack construct. (C) Arthroscopic second look done 6 months later showing intact and stable articular cartilage.

The median osteochondral fragment size was 257.5 mm^2^ (range, 54.9-910.6 mm^2^). All fragments were osteochondral in nature with at least a thin layer of calcified cartilage present. Twelve (32.4%) patients were managed by metallic screw fixation (Figure 1), while 25 (67.6%) were managed with bioabsorbable fixation (Figure 2). No significant differences in median size or major length were seen between metallic or bioabsorbable fixation (*P* = .683 and *P* = .400, respectively). Within the bioabsorbable fixation group, 20 patients were treated with suture bridge fixation in isolation or combined with Arthrex poly-l-lactic acid (PLLA) tacks; 4 patients, with bioabsorbable compression screws; and 1, with PLLA tacks in isolation. Eighteen patients treated with suture bridge fixation had constructs made of absorbable suture, while 2 had nonabsorbable sutures. Transosseous tunnels for suture bridge fixation were used in 17 patients, and suture anchors were used in 3 patients. Procedures done concurrently included 7 medial patellofemoral ligament (MPFL) reconstructions, 3 lateral releases, 2 medial plications, 2 tibial tubercle osteotomies (TTOs), 1 partial meniscectomy, and 1 anterior cruciate ligament reconstruction.

The median follow-up time was 9.6 months (range, 2.1-30.4 months). At the latest follow-up, 32 patients were evaluated with radiographs, while 2 had repeat magnetic resonance imaging scans to assess fracture healing. In total, 33 patients exhibited radiographic evidence of osteochondral fragment integration and healing at their final visit. The cohort’s median final extension was 0° (range, 0°-10° hyperextension), while the median flexion was 135° (range, 90°-140°). Only 1 patient in the metallic screw fixation group had a functional range of motion deficit and arthrofibrosis after OCF fixation, MPFL reconstruction, and TTO. No differences in surgical decision-making were appreciated insofar as there were no statistical differences in the rate of mode of fixation for patellar (*P* = .608), lateral condyle (*P* = .285), or trochlear (*P* = .071) lesions.

**Figure 3.**
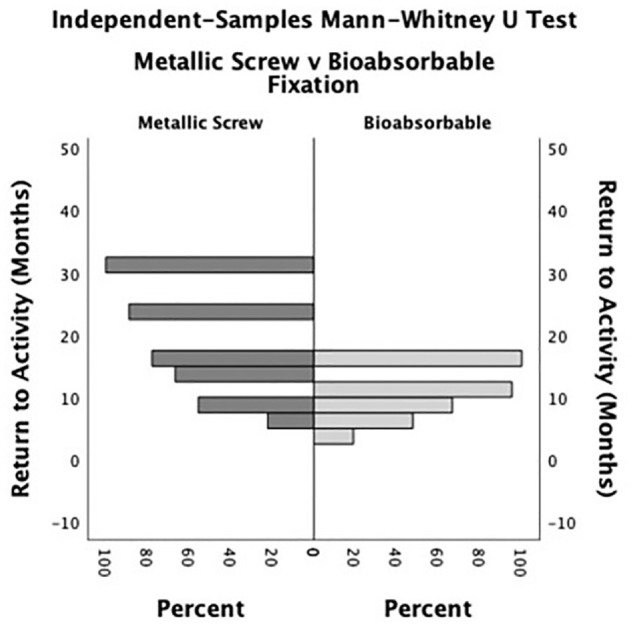
Mann-Whitney *U* test distribution for return to sports and activity time between metallic screw and bioabsorbable fixation (*P* = .04).

The median return to activity time for the entire cohort was 9.1 months (interquartile range, 6.2-12.0 months). No statistically significant differences were appreciated when comparing median time to full activity between the fixation groups (*P* = .427). However, time to full activity was unequally distributed, with 66.7% of the metallic screw fixation group returning to activity later than the total cohort’s median time, compared with 42.9% of the bioabsorbable fixation group (*P* = .04) (Figure 3). At the final follow-up, 8 (66.7%) patients originally treated with metallic screw fixation had reported significant discomfort and returned to the operating room for hardware removal, while no patients with bioabsorbable fixation required return to the operating room (*P* < .001). There were no significant differences between the 2 fixation methods in terms of knee extension (*P* = .865), knee flexion (*P* = .135), radiographic healing (*P* = .235), or complication rate (*P* = .202). However, both complications that arose postoperatively were recorded in the metallic screw fixation group: one patient had an osteochondral malunion but decided against repeat surgical intervention, and the patient previously mentioned with arthrofibrosis eventually had manipulation under anesthesia and lysis of adhesions. Of note, 4 patients had at least 1 noted complaint of mechanical symptoms postoperatively. Three of these were described as “clicking,” and 1 patient noted a sensation of “partial buckling.” Treatment and outcome data are summarized in Table 2.

**Table 2:** Surgical Findings, Treatments, and Outcomes^*a*^

Characteristic	Full Cohort (N = 37)	Metallic Screw (n = 12)	Bioabsorbable Fixation (n = 25)	*P*
Fracture location				
Patella Lateral condyle Trochlea	2 (5) 25 (68) 10 (27)	1 (8) 9 (75) 2 (17)	1 (4) 16 (64) 8 (32)	>.05
Median fragment size, mm^2^	257.5 (54.9 to 910.6)	299.9 (54.9 to 628.0)	243.4 (102.1 to 910.6)	.720
Median fragment diameter, mm	20 (3 to 40)	23 (3 to 40)	20 (13 to 40)	.805
Complications	2 (5)	1 arthrofibrosis (required manipulation under anesthesia) 1 incomplete healing	None	.202
Postop median ROM				
Flexion, deg Extension, deg	135 (90 to 140) 0 (0 to 10)	135 (–90 to 140) 0 (0 to 5)	135 (–90 to 140) 0 (0 to 10)	>.05
Median return to activity, mo	9.1 (3.2 to 30.2)	9.7 (7.0 to 30.2)	7.9 (3.2 to 17.4)	.427
Hardware removal	8 (22)	8 (67)	0 (0)	<.001^*b*^

a Data are presented a n (%) or median (range). Postop, postoperative; ROM, range of motion.

b Statistically significant (*P* < .05).

## Discussion

Overall, good outcomes were seen with high healing and low complication rates regardless of fixation type. Of all the patients included in the study, only one demonstrated inadequate healing and was recommended for fixation revision. Similarly, only 2 patients had complications that warranted a return to the operating room, with one developing arthrofibrosis after MPFL reconstruction and TTO concurrent procedures. Patients treated with bioabsorbable fixation had a significantly different distribution of recovery time than those treated with metallic screw fixation. The reason for the marked difference is rooted in the necessity for hardware removal. Two-thirds of patients treated via metallic screw fixation had secondary procedures to remove symptomatic hardware as compared with 0 patients in the bioabsorbable fixation group. Because undergoing a secondary procedure adds additional weightbearing and activity restrictions, patients who can forgo a return to the operating room are more likely to continue along the recovery path set after their index procedure.

As sports participation rates continue to increase among children and adolescents, PFI and OCFs will continue to be issues commonly faced by the pediatric orthopaedic surgeon. Because of the healing potential associated with the pediatric knee, osteochondral fragment fixation has gained favor as it restores the knee’s native articular cartilage and prevents detrimental sequelae later in life.^13^ Several methods of fixation have been described, with both metallic screw constructs and bioabsorbable fixation constructs showing favorable radiographic and functional results.^8^ Despite the promising results associated with these 2 methods of fixation, no study has directly compared their functional outcomes.

Small-headed metallic screws can provide-long lasting compression to primarily chondral fragments, but they often require a second procedure to be removed and run the risk of being prominent, thereby causing additional damage to opposing cartilage surfaces. Secondary procedures require patients to undergo repeat anesthetic sedation and incur additional financial costs. In a recent study by Rickert et al,^20^ the authors conducted a cost analysis of various methods of medial epicondyle fracture fixation, including screw and suture anchor fixation. The authors found that although suture anchor implants were more costly as individual implants, the overall incurred costs were 10% less than those for screw fixation methods since secondary operation for hardware removal was obviated (total cost of care = $26,650 vs $29,411, respectively). While variation in billing and procedure costs exist between institutions, the additional cost associated with secondary procedures is still enough to produce unnecessary financial hardship, making their results widely generalizable.

Historically, fragment removal and debridement were the gold-standard treatment for traumatic OCFs.^3^ However, recent research has indicated that osteochondral fragment fixation provides better functional outcomes while reestablishing the congruent articular surface of the knee.^13^ Several fixation methods have been described, including both metallic and bioabsorbable constructs, with the aim of providing compression and rotational stability to facilitate range of motion.^8,10^ Bioabsorbable implants offer the advantage of not usually requiring removal; however, the ability of these implants to stabilize the mostly chondral fragments and the time frame over which this stability can be conferred are less than those with metal screws. Absorbable suture bridge constructs can provide compression but depend on fragment congruity to control shear forces. Small bioabsorbable pins and headless biocompression screws provide good resistance to shear force but far less compression than a headed screw.^12,14,15^ Twenty patients in the present study’s cohort were treated with suture bridge fixation, a novel fixation technique that has grown in popularity over recent years.^5,9,15,17^ In a recent 2-patient case series by Bowers and Huffman,^5^ the authors demonstrated good radiographic and functional outcomes with both patients having complete osteochondral union and returning to their baseline level of function 2 years after their initial procedure. Similarly, Vogel et al^25^ described an osteochondral fixation technique utilizing suture anchors. The authors theorized that the use of extra-articular suture anchors is superior to transosseous suture passing methods as it decreases the risk of fractures occurring while drilling the bone tunnel.^25^ Both methods of suture fixation were represented within our study with no discernable differences between transosseous tunnel or suture anchor mechanisms. While larger numbers may be necessary to appreciate any true differences, the findings imply that similar results can be expected regardless of surgeon preference.

### Limitations

This study’s results must be viewed in the context of its inherent limitations. As a retrospective study, it was not impervious to selection bias. Inconsistencies in billing and documentation implies the possibility of not including all eligible patients. In efforts to circumvent this possibility, broad CPT codes were utilized, and records were reviewed by hand to optimize the number of patients in the study. Although patients were divided and analyzed based on the primary method of osteochondral fixation, the study’s sample size was too limited to assess differences across different types of bioabsorbable suture fixation. Additionally, patients in the bioabsorbable fixation group outnumbered those in the metallic screw fixation group 2-fold. Differences in outcome may be exaggerated given this discrepancy; however, the inclusion of all eligible patients was done in an effort to mitigate this bias. Larger and more homogeneous multicentered studies may better be able to assess the differences between these types of bioabsorbable fixation while accounting for other potential confounders. Another limitation was the limited follow-up. Although several patients were seen beyond 12 months and had similar rehabilitation protocols, the median follow-up time of the entire cohort was 9 months, which limited our conclusions regarding chondral healing in the entire cohort. Finally, composite patient-reported outcome measures such as International Knee Documentation Committee scores or Knee injury and Osteoarthritis Outcome Scores were not obtained at each patient visit, making it difficult to compare the complete functional profile between each fixation group. Nevertheless, we believe reporting on postoperative range of motion as well as return to activity time provides a means to compare the short- and midterm outcomes associated with each fixation method.

## Conclusion

Our study is one of the first to directly compare the outcomes between metallic and bioabsorbable osteochondral fixation methods. Good outcomes were seen regardless of fixation type, with high healing and low complication rates. Because of the high rate of secondary hardware removal procedures, metallic screw constructs delayed the return to sports and activity time. Patients treated with bioabsorbable fixation did not require a secondary operation for hardware removal and thus were more likely to recover sooner. Future studies are necessary to assess the potential outcome differences between different types of bioabsorbable fixation methods. Based on these findings, surgeons can consider bioabsorbable fixation as an option for the management of OCF lesions.
